# Modulation of calcium signaling depends on the oligosaccharide of GM1 in Neuro2a mouse neuroblastoma cells

**DOI:** 10.1007/s10719-020-09963-7

**Published:** 2020-11-17

**Authors:** Giulia Lunghi, Maria Fazzari, Erika Di Biase, Laura Mauri, Sandro Sonnino, Elena Chiricozzi

**Affiliations:** Department of Medical Biotechnology and Translational Medicine, University of Milano, Segrate, Milano Italy

**Keywords:** GM1 ganglioside, GM1-oligosaccharide, TrkA neurotrophin receptor, Calcium signaling, Plasma membrane signaling, Neurodifferentiation

## Abstract

Recently, we demonstrated that the oligosaccharide portion of ganglioside GM1 is responsible, via direct interaction and activation of the TrkA pathway, for the ability of GM1 to promote neuritogenesis and to confer neuroprotection in Neuro2a mouse neuroblastoma cells. Recalling the knowledge that ganglioside GM1 modulates calcium channels activity, thus regulating the cytosolic calcium concentration necessary for neuronal functions, we investigated if the GM1-oligosaccharide would be able to overlap the GM1 properties in the regulation of calcium signaling, excluding a specific role played by the ceramide moiety inserted into the external layer of plasma membrane. We observed, by calcium imaging, that GM1-oligosaccharide administration to undifferentiated Neuro2a cells resulted in an increased calcium influx, which turned out to be mediated by the activation of TrkA receptor. The biochemical analysis demonstrated that PLCγ and PKC activation follows the TrkA stimulation by GM1-oligosaccharide, leading to the opening of calcium channels both on the plasma membrane and on intracellular storages, as confirmed by calcium imaging experiments performed with IP3 receptor inhibitor. Subsequently, we found that neurite elongation in Neuro2a cells was blocked by subtoxic administration of extracellular and intracellular calcium chelators, suggesting that the increase of intracellular calcium is responsible of GM1-oligosaccharide mediated differentiation. These results suggest that GM1-oligosaccharide is responsible for the regulation of calcium signaling and homeostasis at the base of the neuronal functions mediated by plasma membrane GM1.

**Supplementary Information:**

The online version contains supplementary material available at 10.1007/s10719-020-09963-7.

## Introduction

Among gangliosides, a particular class of sialic acid-containing glycosphingolipids enriched in neuronal membranes, particular attention is given to GM1, β-Gal-(1–3)-β-GalNAc-(1–4)-[α-Neu5Ac-(2–3)]-β-Gal-(1–4)-β-Glc-(1–1)-Cer (II^3^Neu5Ac-Gg_4_Cer) by virtue of its relevant implication in neuronal differentiation and in neuronal recovery and protection [1–6]. Component of all mammalian brains, GM1 is inserted into the outer layer of the plasma membrane (PM) with the hydrophobic moiety, the ceramide, while the saccharide portion protrudes into the extracellular milieu interacting with a wide range of membrane-associated proteins, including receptors and enzymes [1–6]. It has been extensively studied how GM1 induction of neurite outgrowth and neuroprotective phenomena are accomplished through GM1 specific interaction with neurotrophin tyrosine kinase receptors (Trk) and via its ability to modulate cellular calcium (Ca^2+^) levels, acting on Ca^2+^ influx channels, Ca^2+^ exchangers, and various Ca^2+^-utilizing enzymes [3, 7, 8]. Elevation of endogenous PM GM1 or exogenous applied GM1 in mouse neuroblastoma cells Neuro2a (N2a) and in other neuroblastoma cells leads to the increase of intracellular Ca^2+^, which is accompanied by neurite outgrowth and extension [7, 9, 10]. Additionally, cultured neurons from the B*4galnt1* knock out mouse, characterized by deficiency of GM1 and its oligosialo derivatives, showed impaired Ca^2+^ regulatory capability, which translates in significantly retarded outgrowth [11, 12] and in increased vulnerability to KCl and glutamate excitotoxicity, which is rescued after GM1 application [12].

Although the numerous properties of GM1 have been extensively studied over the years, its mechanism of action is still being explored. It has been proved that GM1-oligosaccharide, β-Gal-(1–3)-β-GalNAc-(1–4)-[α-Neu5Ac-(2–3)]-β-Gal-(1–4)-Glc (OligoGM1; II^3^Neu5Ac-Gg_4_), exogenously added to the culture medium of N2a neuroblastoma cells, was able alone to induce the neuritogenesis process by directly interacting with nerve growth factor (NGF)-specific receptor TrkA at the PM, reserving to the ceramide an exclusively anchor and structural role [13]. Subsequently we found that OligoGM1 administered to cerebellar granule neurons enhanced neuron clustering, neurite sprouting and networking, thus confirming the specific role of the oligosaccharide chain in the processes of neuronal differentiation and maturation, known to be regulated by the entire GM1 [14]. Additionally, the OligoGM1 was found to mediate also the neuroprotective phenomena attributed to ganglioside GM1, being able to induce protection from 1-methyl-4-phenyl-1,2,3,6-tetrahydropyridine hydrochloride (MPTP) neurotoxicity in N2a neuroblastoma cells [15] acting on mitochondria [16], and to rescue the physical and biochemical defects due to the partial lack of GM1 content in a mouse model of Parkinson’s disease [17].

The present work aims to further investigate the mechanism of action of OligoGM1, as the bioactive component of ganglioside GM1, focusing on its ability to modulate the cell Ca^2+^ flow, mechanism at the base of GM1-mediated neuronal differentiation. We describe that the addition of OligoGM1 to the culture medium of N2a cells is able to activate the TrkA-phospholipase C gamma (PLCγ) pathway at the cell surface, leading to an increase of cytosolic Ca^2+^ necessary for OligoGM1-induced cell differentiation. These data contribute to explain the molecular mechanism underlying the neurotrophic action of OligoGM1, shedding new light on the mechanism of action of ganglioside GM1. 

## Methods

### Materials

Commercial chemicals were of the highest purity available, common solvents were distilled before use and water was doubly distilled in a glass apparatus.

Phosphate buffered saline (PBS), sodium orthovanadate (Na_3_VO_4_), phenylmethanesulfonyl fluoride (PMSF), aprotinin, protease inhibitor cocktail (IP), ethylenediamine tetraacetic acid (EDTA), bovine serum albumin (BSA), Xestspongin C, calcium ionophore A23187, mouse anti-alpha-tubulin (RRID: AB_477579) antibody and mouse neuroblastoma N2a cells (RRID: CVCL_0470) were from Sigma-Aldrich (St. Louis, MO, USA). TrkA-inhibitor (CAS 388626-12-8) was from Merk Millipore (Billerica, MA, USA). Fluo-4 acetoxymethyl (AM), 1,2-bis(o-aminophenoxy)ethane-N,N,N′,N′-tetraacetic acid AM (BAPTA-AM), Hank’s Balanced Salt Solution containing calcium and magnesium (HBSS^+^) and Sodium pyruvate were from Thermo Fisher Scientific (Waltham, MA, USA). Rabbit anti-TrkA (RRID: AB_10695253), rabbit anti-phospho-TrkA (Tyr 490) (RRID: AB_10235585), rabbit anti-flotillin-1 (RRID: AB_2773040), and goat anti-rabbit IgG (RRID: AB_2099233) antibodies were from Cell Signaling Technology (Danvers, MA, USA). Mouse anti-PLCγ1 (RRID: AB_628119), anti-phospho-PLCγ1 (Tyr 783) (RRID: AB_2163561), Protein kinase C (PKC) (RRID: AB_628139), anti-phospho-PKCα (Ser 657) (RRID not found) antibodies were from Santa Cruz Biotechnology (Dallas, TX, USA). Mouse anti-calnexin (RRID: AB_397884) antibody was from BD Biosciences. Chemiluminescent kit for western blot was from Cyanagen (Bologna, Italy). 4 − 20% Mini-PROTEAN® TGX™ Precast Protein Gels, Turbo Polyvinylidene difluoride (PVDF) Mini -Midi membrane and DC™ protein assay kit were from BioRad (Hercules, CA, USA). Triton X -100 were from Merk Millipore (Frankfurten, Germany). Cell culture flasks, dishes, and plates were purchased from Corning (Corning, NY, USA). Dulbecco’s modified Eagle’s high glucose medium (DMEM HG), fetal bovine serum (FBS), L-glutamine (L-Glut), Penicillin/streptomycin (10.000 Units/ml) (10.000 U/mL), Streptomycin (10 mg/mL), and acrylamide were purchased from EuroClone (Paignton, UK).

### GM1-oligosaccharide preparation

GM1 ganglioside was purified from the total ganglioside mixture extracted from fresh pig brains collected at the slaughterhouse of the Galbani Company (Melzo, Italy), according to the procedure developed previously [18]. Briefly, high amount of GM1 was obtained by the sialidase treatment of the total pig brain ganglioside mixture. This simplified the purification process as the major part of polysialogangliosides were transformed into GM1 [19]. The ganglioside mixture, 5 g as sialic acid, was dissolved in pre-warmed (36 °C) 500 mL of 0.05 M sodium acetate, 1 mM CaCl_2_ buffer pH 5.5. *Vibrio cholerae* sialidase (1 unit) was added to the solution every 12 h. Incubation at 36 °C and magnetic stirring was maintained for two days, and the solution dialyzed at 23 °C for 4 days against 10 L of water changed 5 times a day. The sialidase treated ganglioside mixture was subjected to 150 cm x 2 cm silica gel 100 column chromatography equilibrated and eluted with chloroform/methanol/water, 60:35:5 by vol. The fractions containing GM1, identified by TLC, were pooled, dried and submitted to a further column chromatographic purification using the above experimental conditions. Fractions containing pure GM1 were collected and dried. The residue was dissolved in chloroform/methanol (2:1 v/v) and precipitated by adding 4 volumes of cold acetone. After centrifugation (15,000 *x* g) the GM1 pellet was separated from the acetone, dried, dissolved in 50 mL of deionized water and lyophilized giving 1,350 mg of white powder which was stored at -20 °C.

The OligoGM1 was prepared by ozonolysis of GM1 followed by alkaline degradation [20] (Supplementary Fig. S1). Briefly, GM1 was dissolved in methanol and slowly saturated with ozone at 23 °C. Triethylamine / water (5:1) was added to the mixture bringing the pH to 10.5–11.0 and the reaction continued for three days. Then, solvent was evaporated and OligoGM1 was purified by flash chromatography using chloroform/methanol/2-propanol/water 60:35:5:5 by vol as eluent. The oligosaccharide was dissolved in methanol and stored at 4 °C.

The NMR spectrum showed a correct ratio between the main peaks and contained no unclear signals, mass spectrometry indicated the correct molecular mass, and HPTLC analyzes performed showed a single band. Overall, and considering that the OligoGM1 was prepared from homogeneous GM1, these results suggest homogeneity for the prepared oligosaccharide (Supplementary Figure S2).

### N2a cell cultures

Murine neuroblastoma cells N2a were cultured and propagated as monolayer on 75 cm^2^ flasks in DMEM HG medium supplemented with 10% heat inactivated FBS, 1% L-glutamine, 1% Penicillin/Streptomycin and 1 mM sodium pyruvate, at 37 °C in a humidified atmosphere of 95% air / 5% CO_2_. Cells were sub-cultured to a fresh culture when growth reached the 80–90% confluence (i.e. every 3–4 days). In sub-cultures passages cells were washed once with PBS and detached by 0.02% EDTA − 0.6% glucose in PBS (w/v).

### Cell treatments

N2a cells were plated at 5 × 10^3^ / cm^2^ on 6-well plates in complete DMEM HG medium for 24 h to allow cells attachment and recovery in complete medium before treatments.

#### OligoGM1 treatment

24 h after plating, growth medium was removed and N2a cells were pre-incubated in pre-warmed (37 °C) DMEM HG medium containing 2% FBS, 1% L-glutamine, and 1% penicillin/streptomycin, for 30 min at 37 °C.

Subsequently, OligoGM1 was solubilized in water at 2 mM concentration by vortex agitation and sonication in water bath 3 times for 30 sec. Solubilized OligoGM1 was administered to cells at the final concentration of 50 µM. This dose condition has been previously found to promote neurodifferentiation and neuroprotection in N2a cells via TrkA-ERK1/2 signaling pathway activation [13, 15]. Control cells were incubated under the same experimental conditions but omitting any addition of OligoGM1.

#### Inhibition of TrkA receptor

To block TrkA activity in N2a cells, TrkA inhibitor (120 nM) was added to the incubation medium 1 h before the addition of OligoGM1 [13, 21].

#### EGTA and BAPTA-AM treatment

To chelate Ca^2+^ ions in N2a cells, the extracellular Ca^2+^ chelating agent EGTA (100 µM) or the intracellular Ca^2+^ chelating agent BAPTA-AM (1 µM) were added to the incubation medium together with OligoGM1 or alone as control condition [22].

### Morphological analysis and neurite outgrowth evaluation

N2a cells untreated (control) or treated with 50 µM OligoGM1 in presence or absence of EGTA/BAPTA-AM for 24 h were observed by phase contrast microscopy (Olympus BX50 microscope; Olympus, Tokyo, Japan).

The neurite-like processes length was measured after treatment with OligoGM1 on bidimensional images acquired with 200X magnification with phase contrast microscopy and expressed as the ratio between neurite length and cell body diameter [13, 23, 24]. Five random fields were examined from each well, giving a total cell count of at least 200 cells per well.

### Isolation of detergent-resistant membrane (DRM) fractions

N2a cells were incubated in the absence (control) or in the presence of 50 µM OligoGM1 for 3 h at 37 °C. Detergent-resistant membrane (DRM) were prepared by ultracentrifugation on discontinuous sucrose gradient of cells subjected to homogenization with 1% Triton X-100, as previously described [25, 26]. Briefly, cells were mechanically harvested in PBS 1X and centrifuged at 270 *x* g for 10 min at 4 °C. Cell pellet was lysed in 1.2 mL of 1% Triton X-100 in TNEV buffer (10 mM TrisHCl pH 10, 150 mM NaCl, 5 mM EDTA pH 7.5) in the presence of 1 mM Na_3_VO_4_, 1 mM PMSF, and 75 mU/mL aprotinin and homogenized for 11-folds with tight Dounce. Cell lysate (2 mg of cell protein/mL) was centrifuged for 5 min at 1,300 *x* g at 4 °C to remove nuclei and cellular debris and obtain a post nuclear supernatant (PNS). A volume of 1 mL of PNS was mixed with an equal volume of 85% sucrose (w/v) in TNEV buffer containing 1 mM Na_3_VO_4_, placed at the bottom of a discontinuous sucrose gradient (30–5%), and centrifuged for 17 h at 200,000 *x* g at 4 °C. After ultracentrifugation, 12 fractions were collected starting from the top of the tube. The light scattering band, corresponding to the DRM fraction, was located at the interface between 5 and 30% sucrose corresponding to fractions 4–6. The entire procedure was performed at 0–4° C on ice immersion. Equal amounts from each fraction were diluted with Laemmli sample buffer (0.15 M DTT, 94 mM Tris-HCl, 3% SDS w/v, 0.015% blue bromophenol, v/v) without glycerol and used for protein analysis as reported below.

### Protein determination

Protein concentration of samples was assessed using a DC™ protein assay kit according to manufacturer’s instructions, using BSA as standard.

### Protein analysis

N2a cells were washed with cold PBS containing 1 mM Na_3_VO_4_ and lysed by hot Laemmli sample buffer (0.15 M DTT, 94 mM Tris-HCl, 15% glycerol, v/v, 3% SDS w/v, 0.015% blue bromophenol, v/v). After the probe sonication (50 W, 30 kHz Vibra-Cell™ Ultrasonic VXC130) and the boiling of the lysed samples for 5 min at 99 °C, equal amounts of denatured proteins derived from OligoGM1 treated and untreated cells were separated on 4–20% precast polyacrylamide gels, and transferred to PVDF membranes using the Trans -Blot® Turbo™ Transfer System (Bio -Rad).

PVDF membranes were blocked with 5% milk (w/v) in TBS-0.1% tween-20 (v/v) at 23 °C for 1 h under gentle shaking. The presence of TrkA and p-TrkA was determined by using specific rabbit primary antibodies, both diluted 1:1,000 in 5% BSA (w/v) in TBS-0.1% tween-20. PLCγ1, P-PLCγ1, PKCα, P-PKCα were detected by the specific mouse primary antibodies diluted 1:500 in 5% milk (w/v) in TBS-0.1% tween-20 (v/v). Flotillin was detected by the specific rabbit primary antibody diluted 1:1,000 in 5% milk (w/v) in TBS-0.1% tween-20 (v/v). Calnexin, used as loading controls, weas detected by the specific mouse primary antibodies diluted 1:40,000 in 5% milk (w/v) in TBS-0.1% tween-20 (v/v) and 1:1,000 in 5% BSA (w/v) in TBS-0.1% tween-20, respectively. The incubation was performed overnight (i.e. 16 h) at 4 °C under gentle shaking. Following, PVDF membranes were washed three times with TBS-0.1% tween-20. The reaction with secondary horseradish peroxidase-conjugated antibodies was following performed at 23 °C 1 h in gentle agitation. The data acquisition and analysis were performed using Alliance Uvitec (Cleaver Scientific Ltd, UK).

### Calcium-imaging

N2a cells were plated at 7.5 × 10^3^ / cm^2^ on a 24 mm coverglass in complete DMEM HG medium. 48 h after plating medium was removed and cells were rinsed three times with HBSS containing calcium and magnesium (HBSS^+^). After washing, cells were incubated with 2.5 µM Fluo-4 AM (494/506 nm) in HBSS^+^ for 30 min at 23 °C in the dark. Subsequently cells were washed three times with HBSS^+^ to remove any dye that is not specifically associated with the cell surface and then incubated in HBSS^+^. Fluorescent emission was examined by live cell analysis using Axio Observer (Zeiss Axio Observer.Z1 with Hamamatsu EMCCD 9100–02) with 400X magnification. The frames were acquired every 5 sec for 20 min (Supplementary Fig. S3). After 3 min of acquisition, 50 µM OligoGM1 solubilized in HBSS^+^ was administered to the cells and after 15 min the Ca^2+^ ionophore A23187 (2 µM) in HBSS^+^ was added to the cells. Control cells were subjected to the same experimental conditions but HBSS^+^ alone without OligoGM1 was administered. Only ionophore responsive cells were analysed. At least 6 cells for field were quantified. The fluorescence for each acquisition (F) was related to the basal fluorescence (Fmin) according to the following formula:

$$ \frac{F- Fmin}{Fmin} $$

To evaluate the involvement of TrkA receptor in the induction of Ca^2+^ influx by the OligoGM1, the TrkA inhibitor (120 nM) was added to the growth medium 30 min before the incubation with Fluo-4. After 30 min, cells were washed three times with HBSS^+^ and then incubated with Fluo-4 AM in HBSS^+^ containing 120 nM TrkA inhibitor for 30 min at 23 °C in the dark. Subsequently, cells were washed three times with HBSS^+^ and incubated in HBSS^+^ with 120 nM TrkA inhibitor.

To inhibit the inositol trisphosphate (IP_3_)-receptors, the selective membrane-permeable inhibitor of IP_3_ receptor Xestospongin C (2.5 µM) [27, 28] was administered together with Fluo-4 for 30 min. After washing with HBSS^+^, Xestospongin C was added again to the working solution and left for the entire duration of the experiment.

### Statistical analysis

Data are expressed as mean ± SEM. The analysis was performed with Prism software (GraphPad Software, Inc. La Jolla, CA, USA). The normality distribution was verified using Kolmogorov–Smirnov, D’ Agostino & Pearson and Shapiro-Wilk tests; in case of a non-Gaussian distribution of data, non-parametric tests were used as indicated in the legend of the figures. A p-value < 0.05 was considered significant.

### Other analytical methods

NMR spectra were recorded with a Bruker AVANCE-500 spectrometer at a sample temperature of 298 K. NMR spectra were recorded in CDCl3 or CD3OD and calibrate using the TMS signal as internal reference. Mass spectrometric analysis were performed in positive or negative ESI-MS. Mass spectra were recorded on a Thermo Quest Finningan LCQTM DECA ion trap mass spectrometer, equipped with a Finnigan ESI interface; data were processed by Finnigan Xcalibur software system. All reactions were monitored by TLC on silica gel 60 plates (Merck).

## Results

### OligoGM1 neuritogenic effect depends on calcium levels modulation

Exogenously administered GM1, or its endogenous increase, induces the differentiation of murine neuroblastoma cells as well as the maturation state of primary neurons [3, 29]. Importantly, the GM1 mediated neurite outgrowth is known to be strictly dependent on Ca^2+^ influx [30–32].

It has recently been observed that the administration of the GM1-oligosaccharide component alone induces the same neuritogenic action in N2a cells, demonstrating that GM1 pentasaccharide is responsible for this effect [13, 14].

Additionally, proteomic analysis of N2a cells exposed to OligoGM1 identified the *ex-novo* expression of several proteins [15] involved in the regulation of Ca^2+^ homeostasis and in Ca^2+^-dependent differentiative and neuroprotective pathways (Table 1), suggesting a possible modulation of Ca^2+^ signaling by OligoGM1.

**Table 1:** List of the Ca^2+^-related proteins statistically differentially expressed in OligoGM1-treated N2a cells with respect to control cells

Majority protein IDs	Protein names	Gene names
P35546	Proto-oncogene tyrosine-protein kinase receptor Ret	Ret
A0A0A6YX01	Protocadherin beta-6	Pcdhb6
E9Q622	Protocadherin 11 X-linked	Pcdh11x
A0A1L1SQU7	FAT atypical cadherin 1	Fat1
Q8VHP6	Cadherin-related family member 1	Cdhr1
Q99PJ1	Protocadherin-15	Pcdh15
F8WJ23	Hornerin	Hrnr
Q91ZZ3	Beta-synuclein	Sncb
Q8BNY6	Neuronal calcium sensor 1	Ncs1
P10493	Nidogen-1	Nid1
E9Q0N0	Intersectin-1	Itsn1
B2RPV6	Multimerin-1	Mmrn1
Q8C845	EF-hand domain-containing protein D2	Efhd2
Q8K3V4	Protein-arginine deiminase type-6	Padi6
Q704Y3	Transient receptor potential cation channel subfamily V member 1	Trpv1
Q9WTR1	Transient receptor potential cation channel subfamily V member 2	Trpv2
B2RQS1	Striatin-3	Strn3
V9GXI9	Striatin-4	Strn4
P48455	Serine/threonine-protein phosphatase 2B catalytic subunit gamma isoform	Ppp3cc
P63328	Serine/threonine-protein phosphatase 2B catalytic subunit alpha isoform	Ppp3ca
A0A087WQ44	Snf2-related CREBBP activator protein	Srcap
Q9QVP9	Protein-tyrosine kinase 2-beta	Ptk2b

Proteins were selected from the full list reported by Chiricozzi et al. [15] and obtained from three biological replicates of OligoGM1-treated versus control N2a cells. For statistical analysis, a *p*-value ≤ 0.01 by Student’s t-test was considered significant

**Fig. 1:**
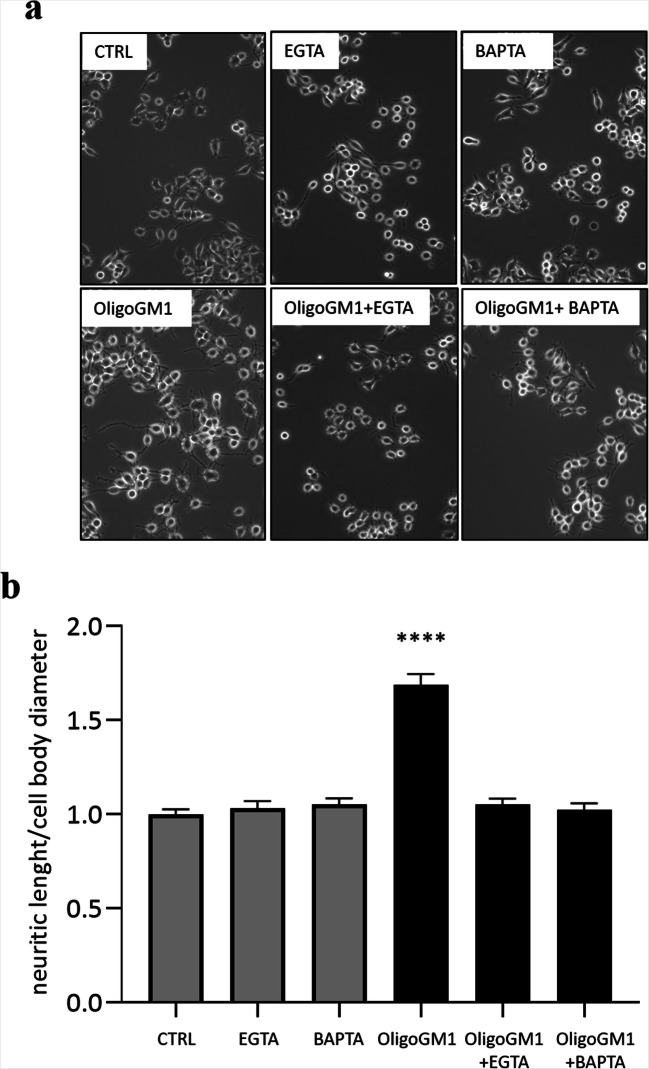
Morphological outcomes of N2a cells treated with 50 µM OligoGM1 in presence or absence of 100 µM EGTA or 1 µM BAPTA-AM for 24 h. **a** Cells were observed by phase contrast microscopy with 200X magnification. Images are representative of three independent experiments (*n* = 3); **b** Evaluation of neurite sprouting and elongation in N2a cells. Neurite extensions were evaluated as the ratio between process length and cell body diameter. The bars show the mean values ± SEM from three different experiments (*n* = 3, OligoGM1 ****p < 0.0001 vs. CTRL, OligoGM1 + EGTA, OligoGM1 + BAPTA, non-parametric one-way ANOVA)

Thus, to study the influence of Ca^2+^ on OligoGM1 enhanced neuritogenesis, 50 µM OligoGM1 was administered to N2a cells in the presence or absence of subtoxic concentrations of extracellular (EGTA, 100 µM) or intracellular (BAPTA-AM, 1 µM) Ca^2+^ chelators for 24 h. None EGTA or BAPTA-AM at indicated concentration affected the N2a cells morphology in our experimental conditions with respect to control N2a cells (Fig. 1).

As highlighted in the images reported in Fig. 1a, the presence of both extracellular and intracellular Ca^2+^ chelators abolished the neurite sprouting induced by OligoGM1 after 24 h. As it emerges from the graph in Fig. 1b, the length of the neuritogenesis in cells incubated with OligoGM1 resulted at least two-fold higher compared to the control cells but equalized the control condition when Ca^2+^ chelators were administered in combination with OligoGM1. This result suggests that, as already reported for GM1 [3], the increase of cytoplasmic Ca^2+^ is fundamental for OligoGM1-mediated neuronal differentiation.

### OligoGM1 modulates intracellular calcium levels

To study the OligoGM1 ability to modulate Ca^2+^ flux required for the neuritogenesis process, we performed calcium-imaging experiments on N2a cells using the non-ratiometric calcium-sensitive Fluo-4 probe. The binding of Fluo-4 to Ca^2+^ results in increased fluorescence excitation at 488 nm and consequently higher fluorescence signal levels.

**Fig. 2:**
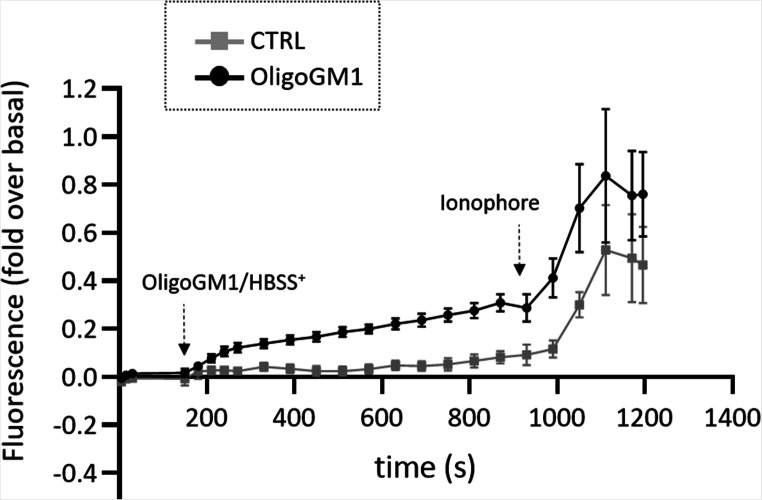
OligoGM1 modulation of intracellular Ca^2+^ level. Intracellular Ca^2+^ level of N2a cells treated with 50 µM OligoGM1 was analysed measuring the green fluorescent emission of 2.5 µM Fluo-4. The frames were acquired every 5 sec for 20 min with Widefields Zeiss Axio Observer.Z1 with a 400X magnification. After 3 min of basal acquisition, OligoGM1 was administered to the cells and after 15 min the calcium ionophore A23187 (2 µM) was added. Control cells were loaded with HBSS^+^ alone. Only ionophore responsive cells were analyzed. The fluorescence (F) of each frame (Fx) was related to the fluorescence of the basal condition (F0) (Fx-F0/F0). Results are expressed as the mean ± SEM of fluorescence intensity of at least three independent experiments (OligoGM1 *p < 0.05 vs. basal, one-way ANOVA, *n* = 11; OligoGM1 **p < 0.01 vs. CTRL, two-way ANOVA; CTRL no significant (NS) vs. basal, one-way ANOVA, *n* = 5)

We find out that the administration of OligoGM1 to undifferentiated N2a cells induces a significant Ca^2+^ influx, starting from about 5 min after OligoGM1 administration as shown in Fig. 2. On the contrary, no increase in fluorescence is observed in control cells. This result proved a direct modulation of intracellular Ca^2+^ flux by the exogenous administration of OligoGM1.

### OligoGM1-mediated calcium modulation depends on TrkA activation

In N2a neuroblastoma cell line, as well as in primary neurons, the OligoGM1 is not internalized by the cells, and its effects are due to a direct interaction with TrkA receptor at the PM level [13, 14]. Thus, taking into account the reported effect prompted by GM1 on TrkA mediated neuronal differentiation [33–38] and considering the recent result where OligoGM1 was found to increase TrkA phosphorylation [13, 14], we investigated if also Ca^2+^ flow modulation could be a molecular event downstream of TrkA activation induced by OligoGM1.

**Fig. 3:**
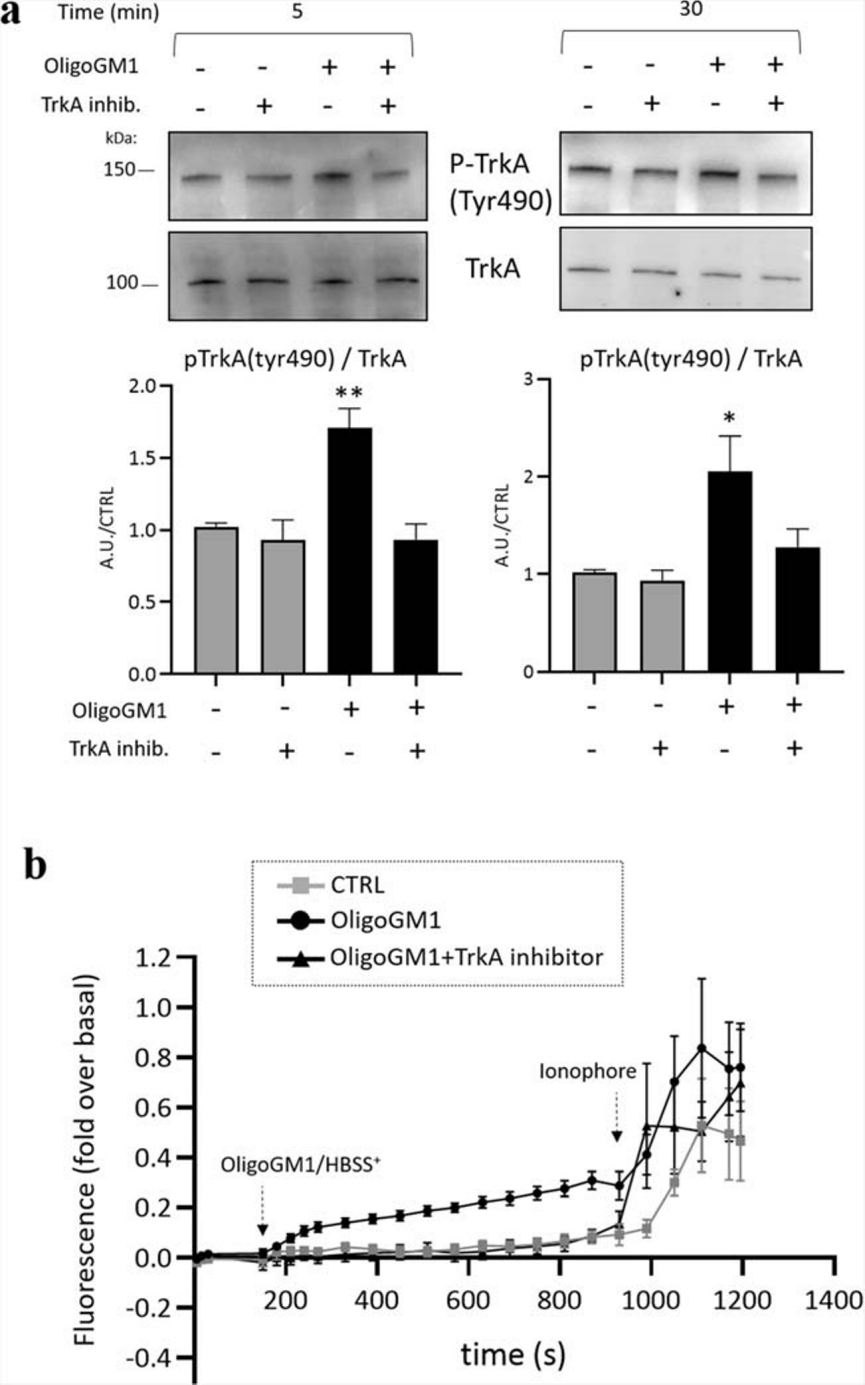
OligoGM1-mediated Ca^2+^ modulation depends on TrkA activation. **a** The TrkA receptor inhibitor (120 nM) was added to the N2a cells 1 h before the administration of 50 µM OligoGM1. Expression of TrkA and p-TrkA (tyrosine 490, Tyr490) was evaluated 5 and 30 min after OligoGM1 treatment by western blot using specific antibodies and revealed by enhanced chemiluminescence. Top: immunoblotting images are shown. Bottom: semiquantitative analysis of p-TrkA related to total level of TrkA. Data are expressed as fold increase over control of the mean ± SEM from three different experiments (*p < 0.05, **p < 0.01, two-way ANOVA, *n* = 3); **b** Intracellular Ca^2+^ level of N2a cells treated with OligoGM1 was analyzed measuring the green fluorescent emission of 2.5 µM Fluo-4. The frames were acquired every 5 sec for 20 min with Widefields Zeiss Axio Observer.Z1 with a 400X magnification. The TrkA inhibitor (120 nM) was added to the culture medium for 30 min before Fluo-4 administration and left for the duration of the experiment. After 3 min of basal acquisition, 50 µM OligoGM1 was administered to the cells and after 15 min the calcium ionophore A23187 (2 µM) was added. Control cells were loaded with HBSS^+^ alone. Only ionophore responsive cells were analyzed. The fluorescence of each frame (Fx) was related to the fluorescence of the basal condition (F0) (Fx-F0/F0). Results are expressed as the mean ± SEM of fluorescence intensity of at least three independent experiments (OligoGM1 * *p* < 0.05 vs. basal, one-way ANOVA, *n* = 11; OligoGM1 **p < 0.01 vs. CTRL, two-way ANOVA; OligoGM1 + TrkA inhibitor NS vs. basal, one-way ANOVA, *n* = 5; OligoGM1 + TrkA inhibitor NS vs. CTRL, two-way ANOVA)

To evaluate the involvement of TrkA receptor, calcium-imaging experiments were performed in the presence of a cell-permeable and highly selective TrkA inhibitor [13, 21]. First, we confirmed that the co-administration of TrkA inhibitor is able to prevent the specific TrkA receptor activation induced by OligoGM1 after 5–30 min from its addition to cell culture medium. (Fig. 3a). Following, we repeated the calcium-imaging experiment in the presence of TrkA inhibitor. As shown in Fig. 3b and Supplementary Figure S3, there is no significant increase of the fluorescence signal in the presence of TrkA inhibitor, suggesting that the opening of the cell Ca^2+^ channels and the Ca^2+^ influx following OligoGM1 administration is mediated by the activation of TrkA receptor.

### Identification of the cellular pathways involved in OligoGM1-mediated calcium influx

To biochemically characterize the effect of OligoGM1 on Ca^2+^ flux in N2a cells following TrkA activation, we investigated the involvement of TrkA downstream effectors known to modulate of Ca^2+^ signaling such as the phospholipase C gamma (PLCγ) and the protein kinase C (PKC) [39].

To unveil if OligoGM1 administration could lead to the increase of intracellular Ca^2+^ through this cellular pathway, an immunoblotting analysis evaluating the phosphorylation status of the TrkA receptor, PLCγ1 and PKCα on N2a cells after 5, 30 and 60 min following OligoGM1 administration was performed.

**Fig. 4:**
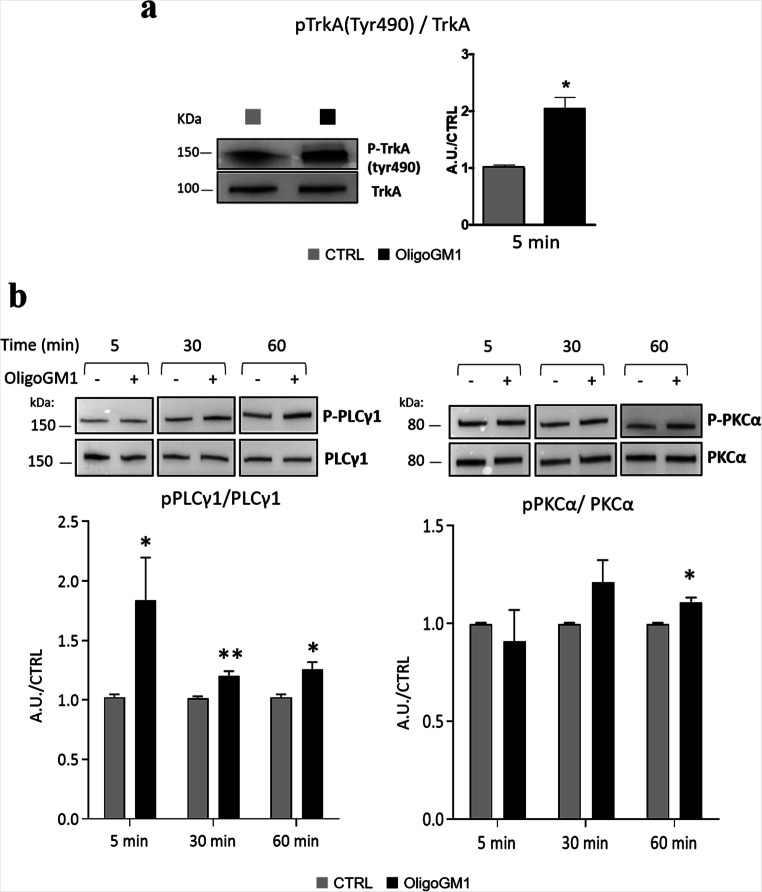
OligoGM1 effect on TrkA-PLCγ1-PKCα pathway. N2a cells were culture in the absence (CTRL) or in the presence of 50 µM OligoGM1. Expression of TrkA, p-TrkA (tyr490), PLCγ1, p-PLCγ1, PKCα and p-PKCα was evaluated 5 min, 30 min and 1 h after OligoGM1 treatment by using specific antibodies and revealed by enhanced chemiluminescence. Top: representative immunoblotting images. Bottom: semi-quantitative analysis of signals of phosphorylated TrkA, PLCγ1 and PKCα related to signals of total TrkA, PLCγ1 and PKCα, respectively. Data are expressed as mean ± SEM of the fold increase over control from at least three experiments (*p < 0.05, **p < 0.01, Student’s t-test)

As shown in Fig. 4 we found that 5 min after OligoGM1 administration there is an activation of TrkA receptor, confirming previous results [13]. Moreover, we observed an enhanced activation of PLCγ1 starting from 5 min from OligoGM1 administration followed, after 1 h, by a hyperphosphorylation of PKCα1, which is a priming event that enables its catalytic activation.

Although the phosphorylation is a key event for the catalytic activity of PKC, it is known that its activation depends on its translocation to the PM [40, 41]. Thus, to verify whether the administration of OligoGM1 was followed by an enrichment of PKC in lipid rafts, 3 h after treating N2a cells with OligoGM1, lipid rafts were isolated as the DRM, according to the procedure described in "Methods" Section.

**Fig. 5:**
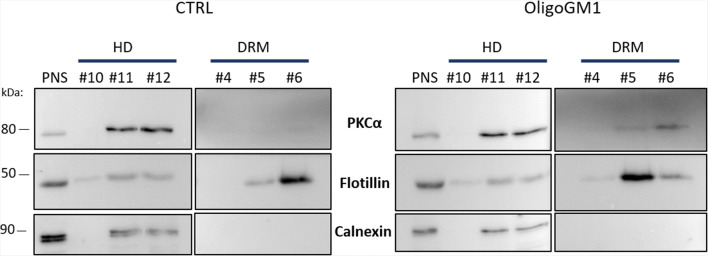
PKCα translocation in lipid rafts microdomains. N2a cells were incubated in the absence (CTRL), or in the presence of 50 µM OligoGM1 for 3 h at 37 °C. Cells were subsequently subjected to sucrose gradient ultracentrifugation to prepare PM microdomains. Twelve fractions were collected from the top of the tube, with fractions 4–6 corresponding to the detergent resistant membrane (DRM) fractions and fractions 10–12 corresponding to high density (HD) fractions. Expression of PKCα, Flotillin (DRM marker) and Calnexin (HD marker) was evaluated by western blot using specific antibodies and revealed by enhanced chemiluminescence. Images are representative of three independent cell culture preparations (*n* = 3)

Western blotting analysis revealed a PKCα enrichment in DRM fractions in OligoGM1 treated cells, while in control cells PKCα is present in the fluid membrane fraction, solubilized by the detergent (Fig. 5).

These events could be responsible for the opening of Ca^2+^ channels on both PM and intracellular storages (i.e. endoplasmic reticulum), resulting in an increase of intracellular Ca^2+^.

**Fig. 6:**
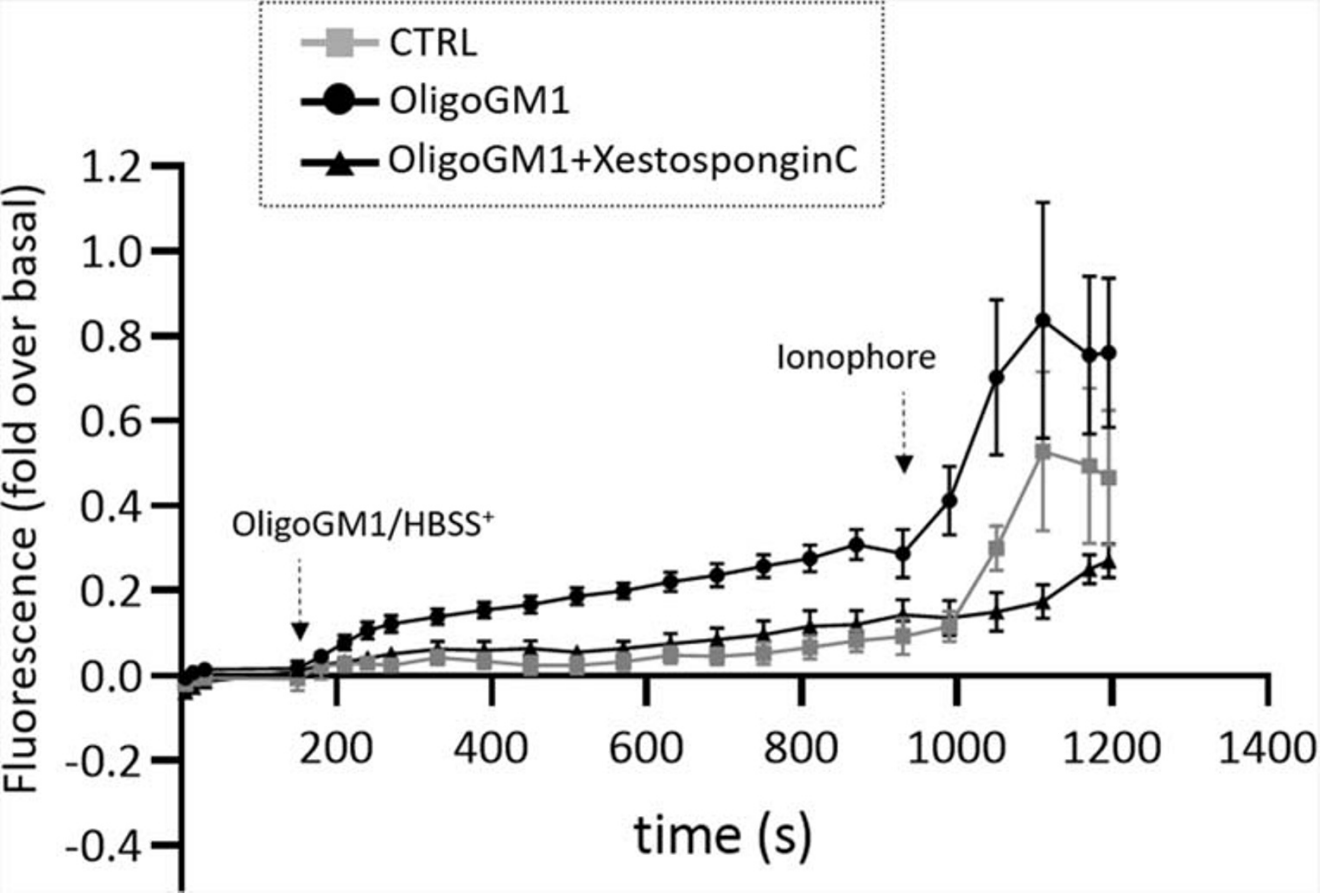
OligoGM1-modulated Ca^2+^ derives from both the extracellular environment and the intracellular storages. Intracellular Ca^2+^ level of N2a cells treated with OligoGM1 was analysed measuring the green fluorescent emission of Fluo-4 (2.5 µM). The frames were acquired every 5 sec for 20 min with Widefields Zeiss Axio Observer.Z1 with a 400X magnification. The IP3 receptor inhibitor, Xestospongin C (2.5 µM) was added to the cells together with Fluo-4 for 30 min before starting the acquisitions and left for the entire duration of the experiment. After 3 min of basal acquisition, OligoGM1 (50 µM) was administered to the cells and after 15 min the calcium ionophore A23187 (2 µM) was added. Control cells were loaded with HBSS^+^ alone. Only ionophore responsive cells were analysed. The fluorescence of each frame (Fx) was related to the fluorescence of the basal condition (F0) (Fx-F0/F0). Results are expressed as the mean ± SEM of fluorescence intensity of at least three independent experiments (OligoGM1 *p < 0.05 vs. basal, one-way ANOVA, *n* = 11; OligoGM1 **p < 0.01 vs. CTRL, two-way ANOVA; OligoGM1 + Xestospongin C *p < 0.05 vs. basal, one-way ANOVA, *n* = 5; OligoGM1 + Xestospongin C NS vs. CTRL, two-way ANOVA)

To disclose if the cytoplasmic Ca^2+^ increase after OligoGM1 administration derives from the extracellular environment through the PM Ca^2+^ channels or from the intracellular storages, the calcium-imaging experiment was performed in the presence of Xestospongin C, a selective, reversible and potent inhibitor of IP_3_ receptors on endoplasmic reticulum [28]. The calcium-imaging representative frames (Supplementary Fig. S3) and the relative graph shown in Fig. 6 demonstrate that in the presence of the IP_3_ receptors inhibitor, the Ca^2+^ influx following OligoGM1 administration is reduced but is not completely abolished, suggesting that both PM and intracellular channels may be modulated by OligoGM1 administration.

## Discussion

For several years ganglioside GM1 has been widely studied for its essential role in neuronal differentiation, protection and restoration [3, 5, 6, 29], accomplished through the cooperation with several players expressed on the PM.

In particular, the GM1 enrichment in PM allows the dimerization and activation of neurotrophins’ receptors belonging to Trk family [3, 42, 43] and modulates Ca^2+^ influx channels and Ca^2+^ exchange proteins causing changes in cellular Ca^2+^ levels [3, 7, 8]. The increase of cytosolic Ca^2+^ is essential for the morphological changes accompanying the neurodifferentiative properties prompted by GM1, triggering specific signaling cascades resulting in actin depolymerization, axon protrusion, and elongation [7, 44–49].

Despite the long-standing research on the GM1, the fine molecular mechanism at the basis of its functions remained obscure until recent years when in a murine neuroblastoma cell line it has been observed that, within the entire molecule, the oligosaccharide chain (OligoGM1) represents, alone, the bioactive component of GM1 ganglioside [13]. Following its isolation from parental compound, the GM1-oligosaccharide added to the culture medium of N2a neuroblastoma cells was observed to induce the neuritogenesis process as equimolar concentration of GM1 did, acting at the PM level by enhancing the phosphorylation of the TrkA receptor followed by the increase in ERK1/2 phosphorylation [13].

This finding was supported by the revelation that, in N2a cells, TrkA and GM1 belong to separate membrane domains. In fact TrkA does not belong to lipid rafts where GM1 is located, suggesting that its interaction with GM1 and the following stabilization which leads to its autophosphorylation involves only the GM1-oligosaccharide chain and the extracellular portion of TrkA, that may flop down on the PM approaching the GM1 saccharide [50]. These results were subsequently translated in a more physiological context by using primary cultures of murine granule cerebellar neurons [14]. According to this study, OligoGM1 administered to primary neurons enhanced cell clustering, neurite sprouting and networking, thus confirming the specific role of the OligoGM1 in the processes of neuronal differentiation and maturation, known to be regulated by the entire GM1.

Here, to further clarify the mechanism of action of OligoGM1, as the bioactive portion of GM1, we examined its ability to modulate the cellular Ca^2+^ flow, at the basis of the neuritogenic properties induced by GM1, using mouse neuroblastoma cells N2a as experimental model.

As shown by calcium-imaging experiments, the administration of 50 µM OligoGM1 to undifferentiated N2a is capable of inducing a significant Ca^2+^ intake starting from 5 min after OligoGM1 application (Fig. 2). This latency time suggests that the entry of Ca^2+^ is not due to the direct interaction of OligoGM1 with Ca^2+^ channels on the PM neither with intracellular channels, since it was demonstrated [13, 14] that the OligoGM1 is not internalized by the cells but rather it could be a result of the activation of PM receptors and downstream signaling pathways.

Since OligoGM1 carries out its neurotrophic and neuroprotective activities interacting with the TrkA receptor on PM, we verified a direct participation of TrkA in the OligoGM1-mediated Ca^2+^ modulation, performing the calcium-imaging experiment in the presence of the TrkA inhibitor. In this case, no Ca^2+^ influx was observed following OligoGM1 administration, indicating that TrkA receptor activation is the upstream event modulating Ca^2+^ flux upon OligoGM1 addition (Fig. 3b).

Subsequently, we studied in more detail the involvement of signaling proteins downstream of TrkA receptor, known to be responsible for cellular Ca^2+^ mobilization, such as PLCγ.

PLCγ is a membrane-associated enzyme that cleaves PIP_2_ into DAG and IP_3_ [51]. The two products of the PLC catalysed reaction, DAG and IP_3_, are important second messengers that propagate and regulate cellular signaling via Ca^2+^ mobilization and activation of protein kinases, such as PKC, and ion channels [51]. When PIP_2_ is cleaved, DAG remains bound to the membrane, whereas IP_3_ is released as a soluble molecule into the cytosol, binding IP_3_-sensitive intracellular Ca^2+^ channels predominately located in the membrane of the endoplasmic reticulum, regulating the Ca^2+^ flux from intracellular stores to the cytosol [52]. The other product, DAG, triggers Ca^2+^ influx from extracellular environment independently from IP_3_ activity by directing plasma membrane TRP channels [53] and activating PKC [54]. The function of the PKC is regulated by two mechanisms: its phosphorylation allows the correct alignment of the residues necessary for the catalysis, while the increase in the intracellular Ca^2+^ concentration triggers the membrane translocation of PKC and its association with DAG at the PM microdomains, stimulating the enzyme activity [40, 41, 54]. Additionally, PKC phosphorylates other molecules, modulating several cellular events: the activation of PKC in the nervous system has been involved in the regulation of ion channels activity, neurotransmitter release, growth, differentiation, and neural plasticity [54].

By immunoblotting analysis we found a hyperphosphorylation of PLCγ occurring 5 min upon OligoGM1 administration (Fig. 4), followed by a hyperphosphorylation of PKC 1 h after OligoGM1 was supplemented to the medium and by its enrichment in lipid rafts, confirming its activation (Fig. 5).

The involvement of IP_3_ in mobilizing Ca^2+^ from the intracellular stores was confirmed by calcium-imaging experiments performed by administering OligoGM1 together with the selective IP_3_ inhibitor, Xestospongin C [27, 28]. Interestingly, while a lower increase in intracellular Ca^2+^ level was recorded (Fig. 6), the Ca^2+^ influx was not completely abolished, suggesting the possible involvement of Ca^2+^ channels of both the intracellular and plasma membranes in the modulation of the cytoplasmic Ca^2+^ levels, which could be activated by IP_3_ and DAG respectively.

**Fig. 7:**
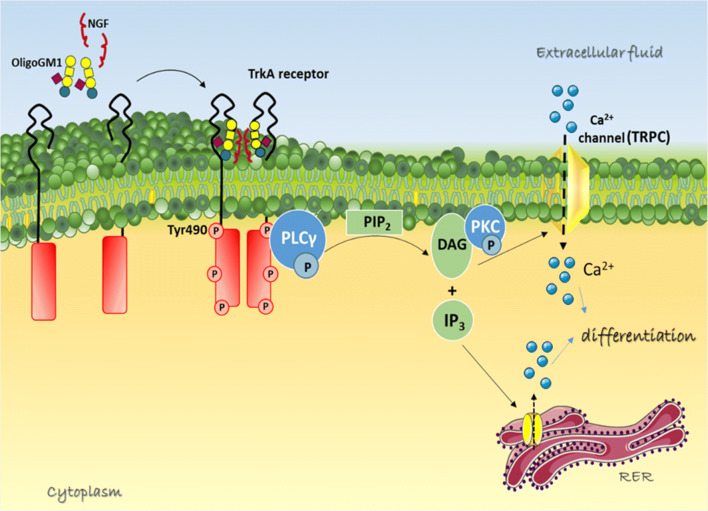
Schematic representation of molecular mechanism underling OligoGM1 neurotrophic function. OligoGM1 enhances the activation of TrkA signaling pathway, which could be associated to an increased activation of PLCγ1, leading to the formation of second messengers DAG and IP3. These events bring to the opening of Ca^2+^ channels on the PM and on endoplasmic reticulum, leading to an increase of cytosolic Ca^2+^ responsible for N2a differentiation. TrkA, neurotrophin tyrosine kinase receptor A; NGF, nerve growth factor; PLCγ, phospholipase C gamma; PIP2, phosphatidylinositol 4,5-bisphosphate; DAG, diacyl-glycerol; IP3, inositol 1,4,5-trisphosphate; PKC, protein kinase C; TRPC, transient receptor potential channel; RER, rough endoplasmic reticulum. GM1 sugar code is according to Varki et al. 2015 [55]. This image is updated from Chiricozzi et al. [13]

Thus, we finally proved that GM1 neuritogenic effect is mediated by an increase in intracellular Ca^2+^ following the OligoGM1-TrkA interaction at the PM level, leading to the recruitment and activation of multiple intracellular players eventually promoting neurite sprouting (Fig. 7). In fact, we observed that OligoGM1 is not able to induce neurite emission in N2a cells if Ca^2+^ ions, both intracellular and extracellular, are chelated (Fig. 1), suggesting that the modulation of cytosolic Ca^2+^ levels by OligoGM1 is fundamental for the execution of its neurodifferentiative properties.

Although here the identity of Ca^2+^ channels modulated by OligoGM1 has not been identified and will be investigate in a later study, the present work demonstrates that the GM1-oligosaccharide is responsible on its own also for the GM1 modulation of Ca^2+^ homeostasis and that the regulation of Ca^2+^ signaling is a fundamental mechanism at the base of OligoGM1 neurogenic action, partially revealing the mechanism of action of the oligosaccharide chain of ganglioside GM1.

## Electronic supplementary material

ESM 1 (PDF 271 kb)

## Abbreviations

A.U.: arbitrary units
BAPTA-AM: 1,2-bis(o-aminophenoxy)ethane-N,N,N′,N′-tetraacetic acid (acetoxymethyl ester)
BSA: bovine serum albumin
Ca^2+^: calcium
CTRL: control
DMEM HG: Dulbecco’s modified Eagle’s high glucose medium
DRM: Detergent-resistant membrane
DTT: 1,4-Dithiothreitol
EDTA: ethylenediamine tetraacetic acid
EGTA: ethylene glycol tetraacetic acid
ERK1/2: extracellular signal-regulated protein kinases 1 and 2
FBS: fetal bovine serum
F: fluorescence
GM1: II^3^Neu5Ac-Gg_4_Cer, β-Gal-(1–3)-β-GalNAc-(1–4)-[α-Neu5Ac-(2–3)]-β-Gal-(1–4)-β-Glc-Cer
HBSS^+^: Hank’s Balanced Salt Solution containing calcium and magnesium
HPTLC: High-performance thin-layer chromatography
IP_3_: inositol trisphosphate
N2a: Neuro2a cells
NS: no significant
OligoGM1: GM1-oligosaccharide, II^3^Neu5Ac-Gg_4_
PBS: phosphate-buffered saline
P-ERK1/2: phosphorylated ERK1/2
PKC: Protein kinase C
PLCγ: Phospholipase C γ
PNS: post nuclear supernatant
P-TrkA: phosphorylated TrkA
PM: plasma membrane
PVDF: polyvinylidene difluoride
RRID: Research Resource Identifiers
TBS: Tris-buffered saline
TLC: thin-layer chromatography
Trk: neurotrophin tyrosine kinase receptor
Tyr490: tyrosine 490
