# Supplementary material for: Modulation of calcium signaling depends on the oligosaccharide of GM1 in Neuro2a mouse neuroblastoma cells
Source: Glycoconj J. 2020 Nov 17;37(6):713–27. doi: 10.1007/s10719-020-09963-7 (PMC7679337; doi:10.1007/s10719-020-09963-7)
Supplement: Supplementary file 1 — (PDF 271 kb) [file 10719_2020_9963_MOESM1_ESM.pdf]

Supporting information

Modulation of calcium signaling depends on the oligosaccharide of GM1 in Neuro2a mouse neuroblastoma cells

Giulia Lunghi*, Maria Fazzari, Erika Di Biase, Laura Mauri, Sandro Sonnino and Elena Chiricozzi*

Department of Medical Biotechnology and Translational Medicine, University of Milano, Segrate, Milano, Italy.

* Correspondence to:

Elena Chiricozzi, PhD

Giulia Lunghi

Via Fratelli Cervi 93, 20090 Segrate (MI), Italy

Phone: +39 0250330381

Fax: +39 0250330365

elena.chiricozzi@unimi.it

giulia.lunghi@unimi.it

Running title:

GM1 in calcium signalling

ORCID ID

Elena Chiricozzi 0000-0001-7431-920

Giulia Lunghi 0000-0002-3099-3395

Methods

Materials

2-propanol, Formic acid, 3-(4,5,-dimethylthiazole-2yl)-2,5-diphenyltetrazolium bromide (MTT) were from Sigma-Aldrich (St. Louis, MO, USA). Hoechst-33342 fluorescent stain was purchased from Thermo Fischer Scientific (Waltham, MA, USA).

Cell authentication

N2a cells are not listed as a commonly misidentified cell line by the International Cell Line Authentication Committee. N2a cells were bought from Sigma-Aldrich to which they were supplied by European Collection of Authenticated Cell Cultures (ECACC) (Catalogue No. 89121404; Lot No. 13K010, passage +9). N2a were used from passage +10 to passage +15 to conduct experiments reported in the present manuscript.

To verify the authentication of employed N2a cells we performed the following tests at the beginning and end of single experimental work.

Morphology check by microscope

To identify the state of cells, we checked cellular morphology by phase contrast microscopy (Olympus BX50 microscope; Olympus, Tokyo, Japan). Morphological outcomes of N2a cells confirmed the expected neuronal/ameboid-like morphology (data not shown).

Growth curve analysis

Cell proliferation was evaluated according to MTT method [1, 2]. Briefly, 2.4 mM MTT (4 mg/mL in PBS) were added to each well and plates were re-incubated for 4 h at 37°C. Medium was carefully removed and replaced with 2-propanol: formic acid, 95:5 (v/v). Plates were gently agitated prior to read the absorbance at 570 nm with a microplate spectrophotometer (Wallac 1420 VICTOR2™, Perkin Elmer). The growth profile showed a normal growth rate (data not shown).

Mycoplasma detection

Mycoplasma infection was evaluated by fluorescent Hoechst staining [3], a fluorescent dye that binds specifically to DNA and that reveals the presence of mycoplasma infections as extracellular particulate or filamentous fluorescence at 400X magnification using NikonEclipse Ni upright microscope. The mycoplasma test has always given negative results (data not shown).

Figure Legends

Fig. S1 Chemical synthesis of GM1 oligosaccharide. Ozone-alkali fragmentation procedure was used to obtain desphingosino-gangliosides. Ozonolysis organic reaction cleaved unsaturated bond between sphingosine 4 and 5 carbons. Subsequently, the alkaline degradation with triethylamine (TEA) released the oligosaccharide chain form the residue. OligoGM1 was purified from the reaction mixture using standard chromatographic procedures and its purity was proved by HPTLC, NMR and mass spectrometry analysis, as shown in Fig. S2. GM1 sugar code is according to Varki *et al.* 2015 [4]

Fig. S2 a) HPTLC representative images. TLC were developed with the solvent system chloroform/methanol/KCl 50 mM, 30:50:13 by vol. Two amounts of OligoGM1 (a,b), Erlich colorimetric revealing method; **b**) 500 MHz 1H-NMR spectrum in DMSO-d6 at 303K of OligoGM1; **c**) Mass spectrometry profile ESI-MS (negative-ion mode): m/z= 997 $[M - H]^-$.

Fig. S3 Representative frames of Fluo-4 emission of basal condition and cell response to OligoGM1 or $HBSS^+$ and after ionopohore administration.

Fig. S1

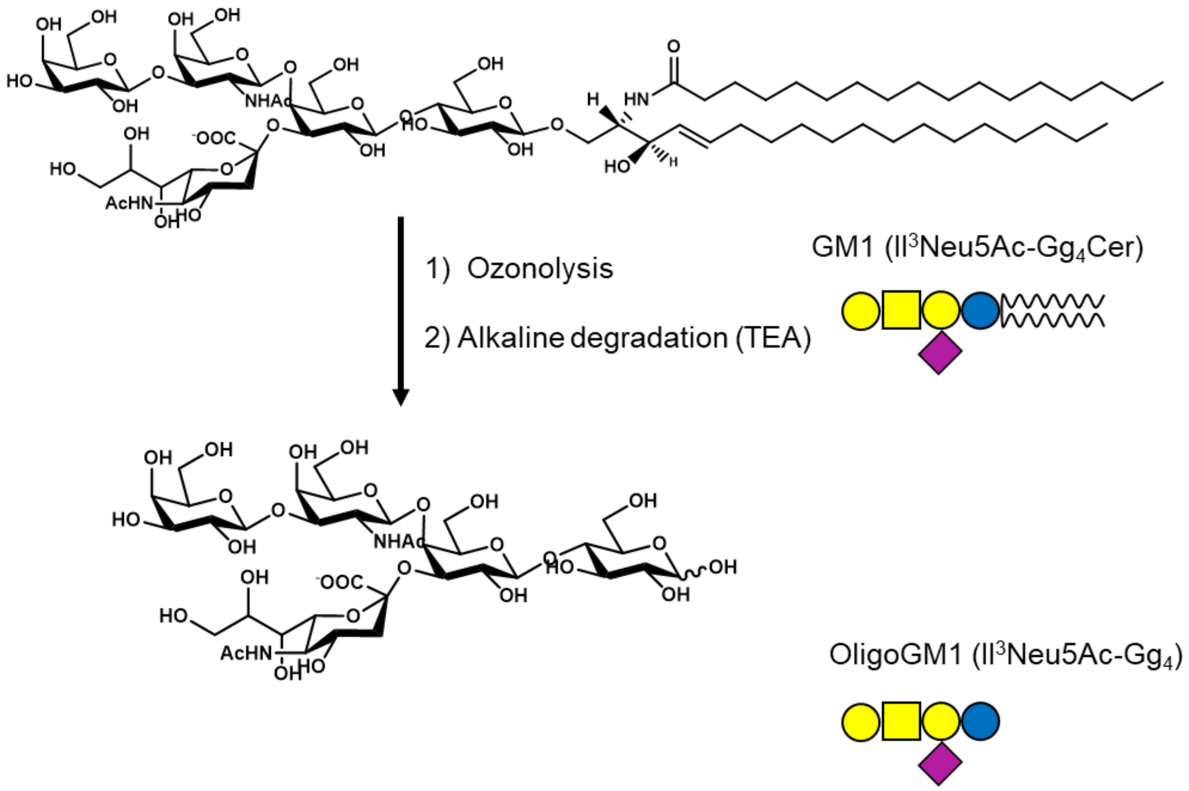
1) Ozonolysis
2) Alkaline degradation (TEA)
GM1 (II^{3}Neu5Ac-Gg$_4$Cer)
OligoGM1 (II^{3}Neu5Ac-Gg$_4$)

Fig. S2

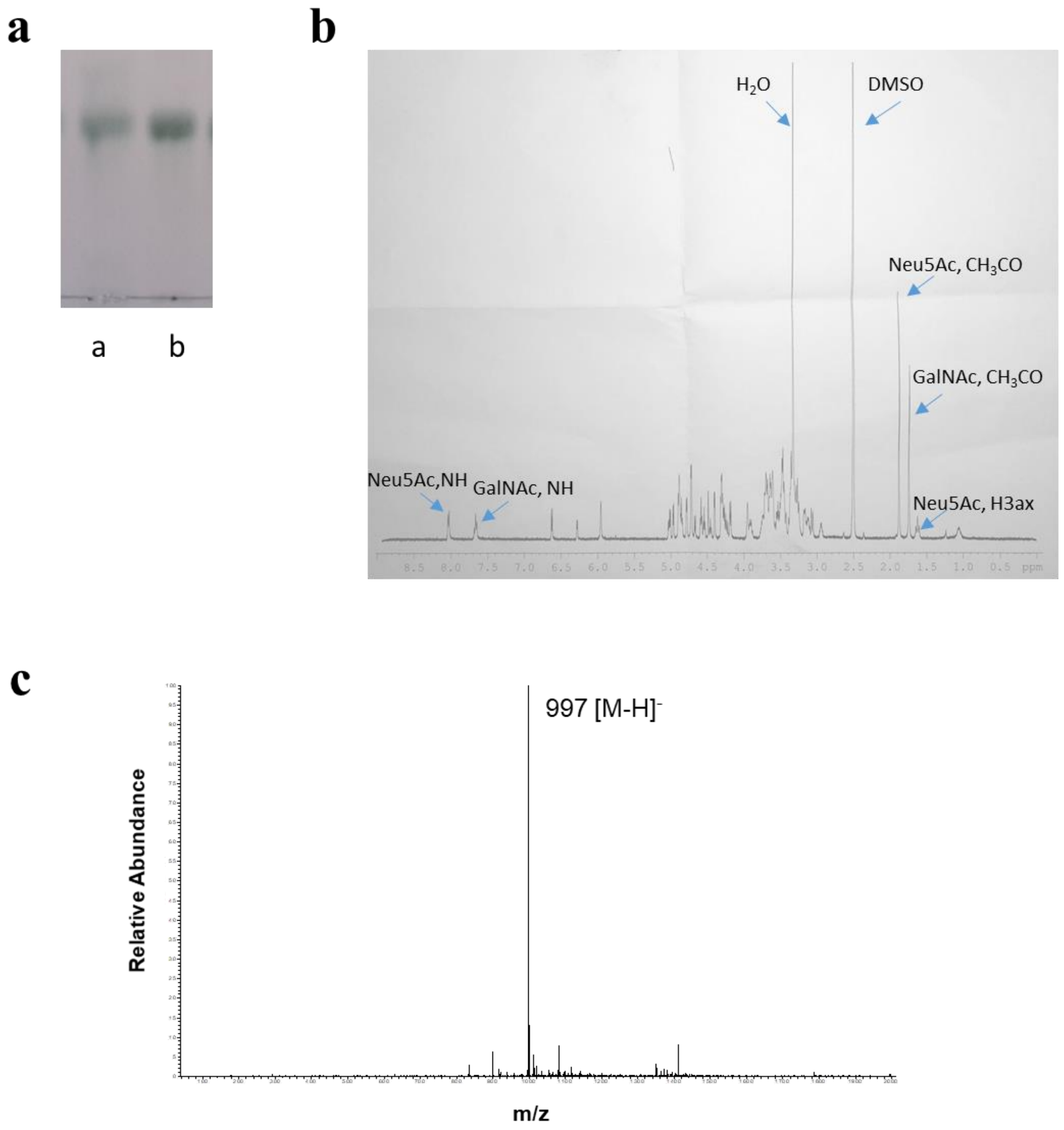
a
b
a
b
H$_2$O
DMSO
Neu5Ac, CH$_3$CO
GalNAc, CH$_3$CO
Neu5Ac,NH
GalNAc, NH
Neu5Ac, H3ax
ppm
c
997 [M-H]$^-$
Relative Abundance
m/z

Fig. S3

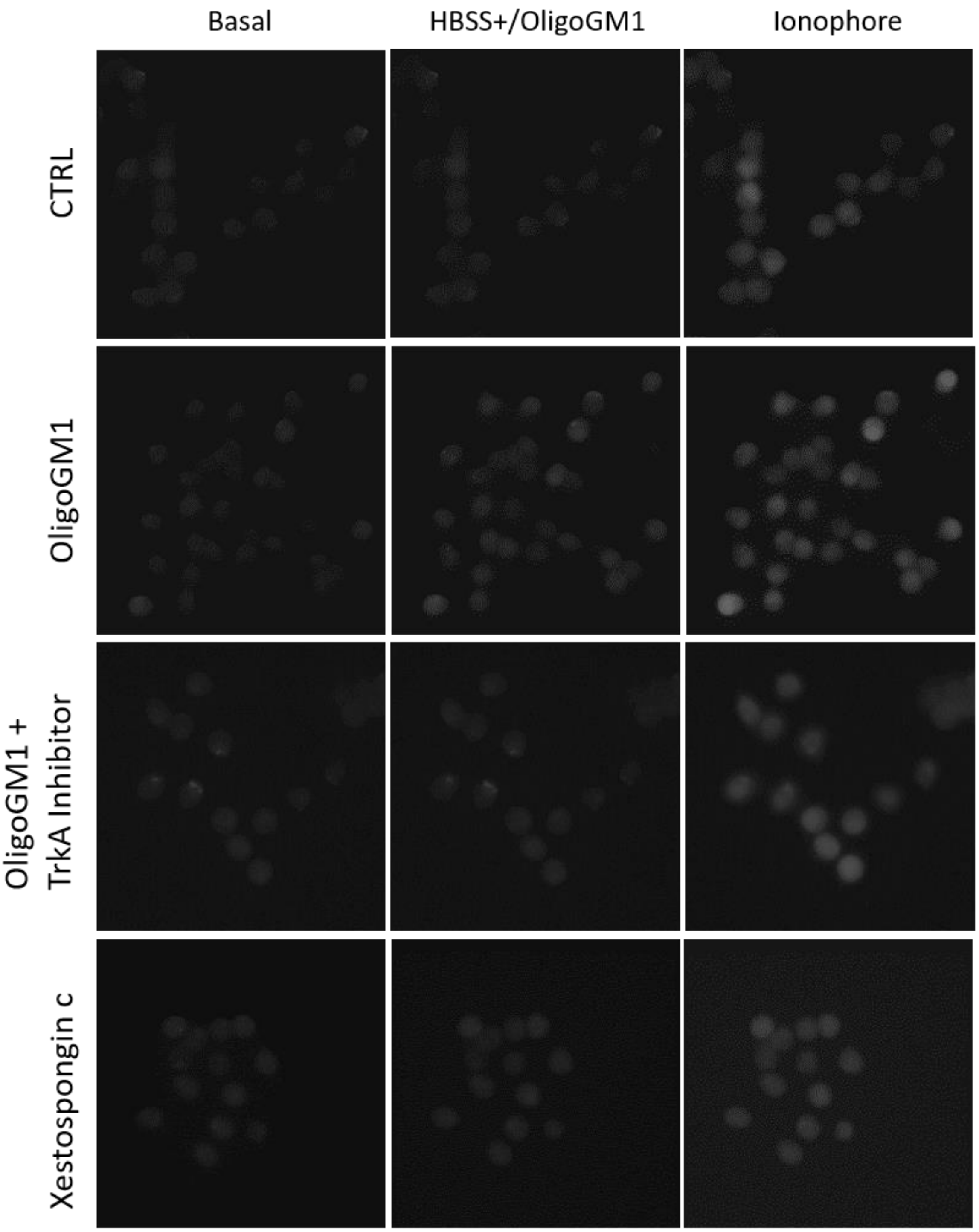

Basal
HBSS+/OligoGM1
Ionophore
CTRL
OligoGM1
OligoGM1 +
TrkA Inhibitor
Xestospongin c

References

1. Mosmann, T.: Rapid colorimetric assay for cellular growth and survival: application to proliferation and cytotoxicity assays. J Immunol Methods. 65, 55-63 (1983).
2. Chiaretti, S.; Astro, V.; Chiricozzi, E.; de Curtis, I.: Effects of the scaffold proteins liprin-alpha1, beta1 and beta2 on invasion by breast cancer cells. Biol Cell. 108, 65-75 (2016).
3. Aureli, M.; Bassi, R.; Prinetti, A.; Chiricozzi, E.; Pappalardi, B.; Chigorno, V.; Di Muzio, N.; Loberto, N.; Sonnino, S.: Ionizing radiations increase the activity of the cell surface glycohydrolases and the plasma membrane ceramide content. Glycoconj J. 29, 585-97 (2012).
4. Varki, A.; Cummings, R.D.; Aebi, M.; Packer, N.H.; Seeberger, P.H.; Esko, J.D.; Stanley, P.; Hart, G.; Darvill, A.; Kinoshita, T.; Prestegard, J.J.; Schnaar, R.L.; Freeze, H.H.; Marth, J.D.; Bertozzi, C.R.; Etzler, M.E.; Frank, M.; Vliegenthart, J.F.; Lutteke, T.; Perez, S.; Bolton, E.; Rudd, P.; Paulson, J.; Kanehisa, M.; Toukach, P.; Aoki-Kinoshita, K.F.; Dell, A.; Narimatsu, H.; York, W.; Taniguchi, N.; Kornfeld, S.: Symbol Nomenclature for Graphical Representations of Glycans. Glycobiology. 25, 1323-4 (2015).
